# Refractory IgA Nephropathy: A Challenge for Future Nephrologists

**DOI:** 10.3390/medicina60020274

**Published:** 2024-02-05

**Authors:** Vincenzo Di Leo, Francesca Annese, Federica Papadia, Maria Serena Russo, Marica Giliberti, Fabio Sallustio, Loreto Gesualdo

**Affiliations:** Department of Precision and Regenerative Medicine and Ionian Area-Nephrology, Dialysis and Transplantation Unit, University of Bari “Aldo Moro”, 70124 Bari, Italy; f.annese7@studenti.uniba.it (F.A.); f.papadia2@studenti.uniba.it (F.P.); serena.russo94@libero.it (M.S.R.); gilibertimarica@gmail.com (M.G.); fabio.sallustio@uniba.it (F.S.);

**Keywords:** refractory IgA nephropathy, SGLT2 inhibitors, new drugs, clinical trials, fecal microbiota transplantation, complement cascades

## Abstract

IgA nephropathy (IgAN) represents the most prevalent form of primary glomerulonephritis, and, on a global scale, it ranks among the leading culprits behind end-stage kidney disease (ESKD). Presently, the primary strategy for managing IgAN revolves around optimizing blood pressure and mitigating proteinuria. This is achieved through the utilization of renin–angiotensin system (RAS) inhibitors, namely, angiotensin-converting enzyme inhibitors (ACEi) and angiotensin receptor blockers (ARBs). As outlined by the KDIGO guidelines, individuals who continue to show a persistent high risk of progressive ESKD, even with comprehensive supportive care, are candidates for glucocorticoid therapy. Despite these therapies, some patients have a disease refractory to treatment, defined as individuals that present a 24 h urinary protein persistently >1 g after at least two rounds of regular steroids (methylprednisolone or prednisone) and/or immunosuppressant therapy (e.g., mycophenolate mofetil), or who do not tolerate regular steroids and/or immunosuppressant therapy. The aim of this Systematic Review is to revise the current literature, using the biomedical database PubMed, to investigate possible therapeutic strategies, including SGLT2 inhibitors, endothelin receptor blockers, targeted-release budesonide, B cell proliferation and differentiation inhibitors, fecal microbiota transplantation, as well as blockade of complement components.

## 1. Introduction

IgA nephropathy (IgAN) is the most common form of primary glomerulonephritis worldwide. [1] It is characterized by the deposition of IgA in the mesangium, a condition that can result in end-stage renal disease (ESRD or end-stage kidney disease, ESKD) within 20 years for 40% of patients. Nonetheless, accurately predicting which IgAN patients will advance to ESRD remains challenging. In the development of IgAN, a central role is played by immune complexes containing specific O-glycoforms of IgA1. Kidney biopsy remains the gold standard diagnostic test for IgAN, and proteinuria and blood pressure represent the primary modifiable risk factors for disease progression. [2,3]

The Kidney Disease Improving Global Outcomes (KDIGO) guidelines propose a comprehensive and effective supportive treatment plan for IgAN, with exceptions for cases involving nephrotic syndrome or rapidly progressive IgAN. [4,5] This strategy encompasses antihypertensive and antiproteinuric medications and education on lifestyle changes (including weight reduction, exercise, smoking cessation, and dietary sodium restriction). Renin–angiotensin system (RAS) blockade remains the primary treatment, proven to reduce proteinuria and slow disease progression. Patients who present a persistently high risk of progressive ESKD, even with comprehensive supportive care, are candidates for glucocorticoid therapy. In fact, following the guidelines, it is currently defined as “high risk” of IgAN progression proteinuria > 0.75–1 g/day despite > 90 days of optimization supportive therapy. Proteinuria reduction to under 1 g/day is a surrogate marker of improved kidney outcome in IgAN, and reduction to under 1 g/day is a reasonable treatment target. But what happens to patients who have persistently high proteinuria despite steroid therapy?

According to the definition provided by Zhao et al. [6], individuals that present a 24 h urinary protein (24-hUP) > 1 g after at least two rounds of regular steroids (methylprednisolone or prednisone) and/or immunosuppressant therapy (e.g., mycophenolate mofetil) or who do not tolerate regular steroids and/or immunosuppressant therapy and have a 24-hUP > 1 g are defined as affected by “refractory IgAN”. Although this definition is not provided in the KDIGO, it is not uncommon that proteinuria may be persistently greater than 1 g/day following a complete regimen of immunosuppressive therapy, and therefore these patients must undergo other treatments. Here, we want to review the current literature regarding the possible therapeutic strategies to be adopted to treat patients affected by “refractory IgAN”.

## 2. The Paradox of Corticosteroid Therapy

According to the KDIGO guidelines, patients who continue to be at a high risk of progressing to kidney failure, typically characterized by having 24-hUP levels greater than 0.75–1.0 g/24 h despite three months of optimized supportive care, should be considered for immunosuppressive treatment, usually a six-month course of glucocorticoid therapy [7].

Although IgAN is an autoimmune disease, the approach to corticosteroid therapy has changed a lot over time. Indeed, a trial on the use of systemic corticosteroids demonstrated a protective effect on the preservation of renal function with no notable adverse effects during follow-up [8].

On the contrary, intensive supportive care plus immunosuppression in IgAN (STOP-IgAN) was used to investigate whether the addition of corticosteroids/immunosuppressive therapy to comprehensive supportive care would yield better results compared to using supportive care alone in patients with IgAN. 

The primary endpoint, after three years, was full clinical remission or a decrease in the estimated glomerular filtration rate greater than 15 mL/min/1.73 m^2^.

The trial included a six-month run-in period to ensure optimal supportive care before randomization. Eligible patients had a 24-hUP between 0.75 and 3.5 g per day and an eGFR greater than 30 mL/min/1.73 m^2^ after the run-in phase and were randomized into two groups: One receiving supportive care alone and the other receiving supportive care combined with immunosuppression, which included corticosteroids for all, with the addition of cyclophosphamide followed by azathioprine if the eGFR was between 30 and 59 mL/min/1.73 m². This study demonstrated no difference in renal function between the two groups [9,10].

The STOP-IgAN study was followed by the TESTING I study, conducted primarily in a Chinese population using 0.8 mg/kg/day of methylprednisolone. This trial was stopped prematurely due to significant side effects, the most important of which were fatal and life-threatening infections. This has raised fresh concerns regarding the effectiveness of corticosteroids in treating IgAN, leading to the TESTING II trial.

The protocol was adjusted in this last study by reducing the dose of methylprednisolone to 0.4 mg/kg/day (maximum 32 mg/day, weaning by 4 mg/day/month) and adding prophylactic sulfamethoxazole-trimethoprim. Among patients with IgAN at high risk of progression, treatment with oral methylprednisolone for six to nine months, compared to a placebo, significantly reduced the risk of the composite outcome of kidney function decline, kidney failure, or death due to kidney disease. Moreover, the risk of infections was mitigated with a reduction in dosage and the addition of antibiotic prophylaxis with sulfamethoxazole–trimethoprim, such that a lower incidence of serious adverse events was observed in the reduced-dose groups after these changes were made (16% in TESTING I, 5% in TESTING II, and 3% in the placebo) [10].

Hence, while corticosteroids may offer short-term benefits to specific IgAN patient populations, their potential side effects warrant careful consideration. There is a need for alternative approaches that balance effectiveness with safety. According to the KDIGO guidelines, when contemplating the use of systemic corticosteroids for IgAN, special attention should be given to the increased risk of steroid-related adverse effects in patients with an eGFR below 50 mL/min/1.73 m². It is advisable to avoid corticosteroids in patients with an eGFR below 30 mL/min/1.73 m², as well as in those with diabetes mellitus, obesity, untreated latent infections (such as viral hepatitis, tuberculosis, or HIV), active peptic ulcer disease, severe osteoporosis, or uncontrolled psychiatric conditions. Patients considering immunotherapy should undergo thorough screening for chronic infections and receive proper vaccinations to mitigate infectious risks. Furthermore, it is important to exercise caution by limiting both the dosage and duration of steroid treatments [4,11].

## 3. New Drugs for IgAN

Currently, there is a lack of targeted therapy specifically designed for IgAN, given the incomplete understanding of its underlying mechanisms. The primary goal of treating IgAN patients is to slow down the progression of the disease, typically achieved through comprehensive supportive care. 

The literature research was performed using the PubMed bibliographic database. The keyword “refractory IgAN” was used to formulate the search string for the literature evaluation. Our inclusion criteria comprised studies, written in English, concerning humans. We found 73 results in total. Furthermore, we searched in PubMed for “Clinical trials for IgAN” and, using the “clinicaltrials.gov” (accessed on 01 December 2023) site, we selected experimental clinical trials of drugs that could represent potential treatment avenues for subjects suffering from refractory IgAN. 

Although these drugs have not yet been approved for the treatment of patients with refractory IgAN, they offer a promising outlook for addressing this condition in the future [12]. Furthermore, it can be hypothesized that in the future, these drugs could be used in a synergistic manner so as to target multiple disease pathways. Indeed, these various strategies focus on inhibiting signaling pathways, depleting plasma cells, clearing IgA deposits, modulating mucosal immunity, and blocking complement cascades to treat different aspects of IgAN pathogenesis and progression [13,14].

**Figure 1:**
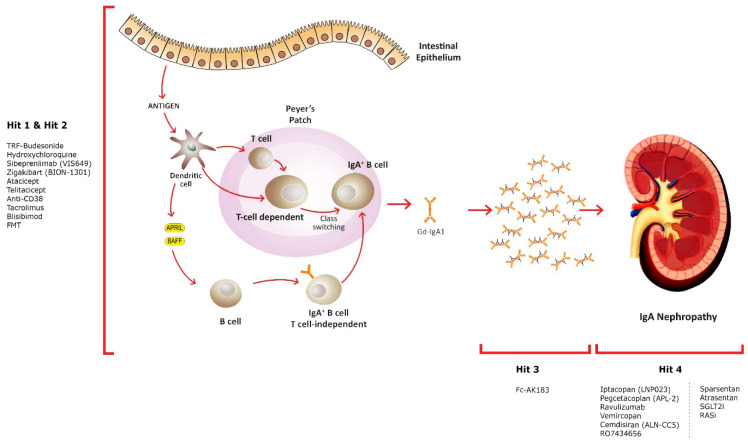
Innovative treatment approaches and their targets.

Emerging therapeutic drugs and their targets are presented in Table 1 and Figure 1.

The hypothesis explaining the pathogenesis of IgAN suggests an elevation in the synthesis of IgA1 with deficient galactose in some O-glycans, serving as an autoantigen. Elevated levels of Gd-IgA1 in circulation (Hit 1) are accompanied by the presence of unique circulating anti-glycan autoantibodies (Hit 2). This interaction gives rise to the formation of pathogenic IgA1-containing circulating immune complexes (Hit 3). Subsequently, these complexes may deposit in the glomeruli, leading to renal injury (Hit 4). 

### 3.1. Supportive Therapy

Numerous emerging supportive strategies are broadening the array of options beyond single RAS blockade therapy. Among these, sparsentan, a dual endothelin–angiotensin receptor antagonist, is being evaluated for its kidney-protective potential in high-risk IgAN patients through the PROTECT trial (NCT03762850). In this study, participants were randomly assigned in a 1:1 ratio to receive 400 mg of sparsentan or 300 mg of irbesartan once daily. The interim results showed a statistically significant reduction in proteinuria in the sparsentan group compared to the irbesartan arm. [15] Moreover, the endothelin A receptor inhibitor atrasentan is also under investigation in the ongoing phase III ALIGN study (NCT04573478) [16].

In the context of supportive care, there are new oral drugs called gliflozins, or sodium–glucose cotransporter-2 inhibitors (SGLT2Is); first used exclusively as antidiabetic medicaments and then considered useful in nephroprotection in diabetic and non-diabetic patients [17]. 

Dapagliflozin, canagliflozin, empagliflozin, and ertugliflozin are currently approved in Europe. In Italy, at present, only dapagliflozin is prescribed for chronic kidney disease (CKD).

Gliflozins are sodium–glucose cotransporter-2 (SGLT2) inhibitors that reduce glucose reabsorption in the proximal convoluted tubule of the kidney, causing glycosuria and natriuresis [18].

SGLT2 transporters are part of the SGLT family, comprising six protein isoforms that mediate the transport of glucose, osmolytes, ions, vitamins, and amino acids. SGLT2s are symports located on the apical membrane of the S1 portion of the proximal tubule cells. They transport glucose and sodium at a 1:1 ratio by active transport against a concentration gradient [19,20]. On the antiluminal side, the reabsorbed glucose leaves the intracellular space by passive diffusion through glucose transporters 1 and 2 (GLUT1 and GLUT2), while sodium is extruded via ATP-mediated active transport [21,22].

The nephroprotective effects of SGLT2Is derive from different mechanisms of action [23]. 

SGLT2Is, promoting glycosuria and natriuresis, improve tubulo-glomerular feedback for the higher sodium load that reaches the macula densa and cause vasoconstriction of afferent arterioles and vasodilation of efferent arterioles. In this way, SGLT2Is reduces intraglomerular pressure and, thus, hyperfiltration [24]. 

These hemodynamic changes within the glomerulus first cause a drop in eGFR and then a stabilization phase. This trend can be well described by a “checkmark” sign (√), which faithfully reflects the mechanism of action of these drugs [25,26].

In diabetic and non-diabetic mice with bovine serum albumin-induced renal injury, dapagliflozin reduced proteinuria, glomeruli damage and dysfunction, and podocyte loss [27].

In addition, by reducing oxygen consumption, normally used for sodium reabsorption, SGLTIs increase renal oxygen tension, contributing to nephroprotection [28].

Moreover, in damaged kidneys, SGLT2Is can reduce the functional overload of healthy remnant nephrons, which, on the contrary, reabsorb a high load of sodium, proteins, and solutes, contributing to metabolic stress on the tubular epithelial cells of CKD kidneys [29]. 

In addition, in a meta-analysis concerning SGLT2Is, it was demonstrated that these drugs reduce the burden of inflammatory molecules that cause oxidative stress and endothelial dysfunction, whose role is known to be implicated in the progression of chronic kidney disease [30,31]. In particular, among pro-inflammatory molecules, IL-6 is of considerable importance, whose reduction seems to play a fundamental role in reducing damage in chronic renal disease, resulting in a reduction in proteinuria and a decrease in deposition of the mesangial matrix within the glomerulus [32].

Physiologically, 180 mg/dL of glucose (the value corresponding to the “true renal threshold” of glucose) is filtered by all nephrons, while in patients with CKD, this occurs at the expense of a smaller number of nephrons. Some studies in the field of diabetes have confirmed that, at the level of the proximal tubule, hyperglycemia-induced pro-inflammatory cytokines are involved in tubular damage [33,34,35]. In a recent in vitro study, it was shown that in diabetic patients, hyperglycemia causes the activation of inflammatory pathways (inflammasome activation and increased TNFα expression), increased TNFα expression, and a reduction in autophagic flux. Dapagliflozin improves these hyperglycemia-induced changes [36]. These considerations could therefore be transferred to non-diabetic CKD, since, in this case, the nephrons are exposed to a higher dose of glucose, not derived from hyperglycemia but from the reduction of functioning nephrons caused by the underlying pathology.

Finally, emphasizing the importance of blood pressure control in patients with IgAN and in general in those with CKD, gliflozins cause a reduction in cardiac preload and afterload through natriuresis and osmotic diuresis, thus improving blood pressure control and vascular dysfunction [37].

In the randomized DAPA-CKD trial, 4304 adults with CKD were divided into groups of dapagliflozin (10 mg/day) vs. placebo. Dapagliflozin reduced the occurrence of ESKD by 36%, cardiovascular event death and the risk of hospitalization for heart failure by 29%, and the decline in kidney function and cardiovascular events [38]. A reduction in the progression of renal failure with proteinuria was seen in people both with and without diabetes. In the DAPA-CKD trial, patients with IgAN were the third largest group after diabetic nephropathy and ischemic/hypertensive nephropathy. Among IgAN patients, dapagliflozin reduced the risk of primary composite outcomes (decline in eGFR ≥ 50%, onset of end-stage kidney disease, or death from a kidney disease-related or cardiovascular cause) by 71% and secondary kidney-specific outcomes (similar to the primary outcome but excluding cardiovascular death) by 75%. Dapagliflozin was well tolerated in the IgAN population, showing its already known safety profile in this group [39].

In the EMPA-KIDNEY trials, which enrolled 6690 patients in the empagliflozin (10 mg/day) vs. placebo arms, the safety of SGLT2Is was also studied in IgAN patients, as was their efficacy in reducing the risk of progression of chronic kidney disease and death from cardiovascular causes [40].

In terms of adverse events, it has been shown that SGLT2Is can cause an increased risk of ketoacidosis and amputation, but this risk is countless times lower than the benefits of this class of drugs, even in patients with diabetes. Gliflozins may increase the risk of genital and urinary infections; these events, which can be contained with accurate personal hygiene and precise medical history collection, should not be considered an absolute contraindication at the beginning of this therapy or its continuation [41].

In conclusion, the nephroprotection induced by SGLT2Is could be considered equal or synergistic with that offered at present by ACEi and ARBs for IgAN patients and, in particular, subjects affected by refractory IgAN.

### 3.2. Modulation of Mucosal Immunity

Given the pivotal role of mucosa-associated lymphoid tissue (MALT), particularly in Peyer’s patches, in the synthesis of Gd-IgA1 molecules, the primary approach to treating individuals with IgA nephropathy (IgAN) focuses on diminishing the production of these altered immunoglobulins. In response to this situation, a novel oral, targeted-release glucocorticoid formulation called TRF-budesonide (NEFECON^®^, Everest Medicine, Hong Kong, China) is now the first fully FDA-approved treatment for patients with IgAN at high risk of progression to kidney failure [42].

This second-generation synthetic corticosteroid is designed to gradually release budesonide, in a targeted formulation, in the terminal ileum, where mucosal Peyer’s patches are most concentrated, causing a downregulation of the local production of Gd-IgA1. In the recent NEFIGAN trial and subsequent NEFIGARD study, a nine-month regimen of once-daily oral Nefecon (16 mg) demonstrated statistically significant reductions in proteinuria, a slowdown in the decline of renal function, and lowered serum levels of the Gd-IgA1 and Gd-IgA1–IgG immune complexes [43].

### 3.3. Modulation of Gd-IgA1 and Immune Complex Production

Regarding the regulation of inflammation-related signaling pathways, the activation of Toll-like receptors (TLRs) and the upregulation of signaling by B cell-activating factor (BAFF) and a proliferation-inducing ligand (APRIL) are closely associated with the survival and function of B cells and plasma cells. Inhibition of excessive TLR activation by hydroxychloroquine (HCQ) has shown potential for protecting the kidneys and reducing inflammation in IgAN patients. Monoclonal antibodies like blisibimod, targeting BAFF and APRIL, have demonstrated efficacy in reducing peripheral B cells, immunoglobulins, and proteinuria in IgAN patients. Anti-APRIL antibodies such as VIS649 (Sibeprenlimab) and BION-1301 (Zigakibart) are being evaluated in clinical studies. Atacicept, a soluble TACI–immunoglobin fusion protein, can inhibit both BAFF and APRIL-mediated B cell class switching, potentially reducing antibody levels. Telitacicept is a fusion protein composed of a transmembrane activator, a calcium-modulating cyclophilin ligand interactor, and a fragment crystallizable portion of immunoglobulin G (IgG), which neutralizes the B lymphocyte stimulator and a proliferation-inducing ligand [44]. Clinical trials are underway to assess the safety and efficacy of these treatments [45].

Therapies involving anti-CD20 antibodies aim to eliminate B cells for long-term IgAN treatment. While these antibodies, like rituximab, effectively deplete B cells, they often fail to eliminate long-lived plasma cells (LLPCs) and the levels of pathogenic antibodies. Strategies targeting CD38 + plasma cells, such as felzartamab (MOR202), a human IgG1 monoclonal antibody, are being investigated for their potential in reducing these cells and related antibodies. Moreover, mezagitamab (TAK-079) is an anti-CD38 human IgG1 monoclonal antibody, and it is under clinical development by Takeda Pharmaceutical and currently in phase I for IgAN (NCT05174221) [46].

Excessive deposits of pathogenic IgA1 and circulating immune complexes (CICs) are targeted for clearance in IgAN treatment. Enhancing the function of IgA receptors and developing potent IgA1 proteases to break down IgA deposits are potential strategies. IgA receptors like Fc_RI (CD89) and CD71 are crucial in IgAN pathogenesis. [47] Bacteria-derived IgA proteases have shown promise in cleaving IgA1 and IgA1-containing immune complexes, potentially reducing mesangial IgA deposits. Recombinant fusion proteins like Fc-AK183, a fusion of IgA protease and human IgG Fc, demonstrate clearance of circulating and deposited IgA in animal models [48].

Another therapeutic approach is represented by calcineurin inhibitors (CNIs), which include tacrolimus (TAC), which is able to suppress the immune response by downregulating the transcription of various genes in T cells [49]. TAC binds to T lymphocyte-specific FK506-binding protein (FKBP) to form the TAC–FKBP12 complex, which then binds to calcineurin and alters the transcription of numerous genes in T cells, thus lowering the immunological response. There is considerable debate regarding TACs general benefits in the treatment of IgAN [50,51].

According to a meta-analysis by Yong Zhang based on 11 studies including 540 patients with IgAN, TAC combined with glucocorticoids is advantageous in lowering proteinuria when compared to the control group. The most frequent adverse effects described include hypertension, issues with liver function, increased serum creatinine, and gastrointestinal discomfort [52].

Based on the findings of a study, TAC accelerated proteinuria remission in individuals with non-rapidly progressing IgAN without increasing the risk of adverse events. This retrospective cohort analysis included 127 patients with a 24-hUP ≥ 1 g/day and ≤3 mg/dL of serum creatinine. In this study, the authors found that tacrolimus effectively reduced proteinuria in patients with non-rapidly progressive IgAN, with a more rapid decline in proteinuria than that observed in the non-tacrolimus group (which included glucocorticoids, glucocorticoids plus cyclophosphamide, glucocorticoids plus mycophenolate mofetil, and mycophenolate mofetil) at 3, 9, and 12 months after the onset of treatment. The total remission (95.1%) and complete remission (62.3%) rates were significantly higher in the tacrolimus group than in the non-tacrolimus group. These results were also observed 9 and 12 months after the onset of treatment [52].

Fourteen patients with refractory IgAN were included in a different study. The patients received tacrolimus (0.05–0.1 mg/kg/day) and prednisone (0.5 mg/kg/day each) for at least six months. Tacrolimus showed rapid proteinuria remission in patients with refractory IgAN [50].

Seven pertinent trials involving 374 patients were carried out as part of a systematic literature review. Proteinuria of less than 0.5 or 0.3 g/day was used to define the complete remission rate. A proteinuria reduction to at least 50% of the baseline value and an absolute value greater than or equal to 0.5 or 0.3 g/day were considered signs of partial remission [53]. The findings of this research revealed that CNIs plus medium/low-dose steroid therapy resulted in a higher complete remission rate when compared to steroid therapy alone or a placebo, but that partial remission rates were not significantly different.

According to these studies, CNI therapy, on the contrary, is linked to a higher incidence of adverse effects, including gastrointestinal problems, nephrotoxicity, and metabolic problems [54].

### 3.4. Inhibitors of Complement Cascades

Complement cascades, specifically targeting the downstream factors of C5, are under investigation in phase II/III trials. [55] Various inhibitors, such as anti-C5a receptor antagonist (CCX168), eculizumab-derived C5-blocking antibody (ravulizumab, ALXN1210), and small interfering RNA-targeting C5 (cemdisiran, ALN-CC5), are being evaluated for their safety and efficacy. Complement-directed therapies targeting alternative pathways are also being explored [56].

Among these, iptacopan (LNP023) is an orally administered targeted factor B inhibitor of the alternative complement pathway. Based on the APPLAUSE-IgAN study (NCT04578834), patients treated with LNP023 had a decrease in proteinuria and a small change in their eGFR compared to the placebo group [57].

Moreover, it should be mentioned that pegcetacoplan (APL-2), which is a PEGylated, lab-made peptide derivative of compstatin that prevents the cleavage and activation of C3 (NCT03453619) [58,59] and vemircopan (ALXN2050), is an oral factor D inhibitor that inhibits the alternative pathway in a near-complete and sustained manner (NCT05047458 and NCT05047484) [60,61].

To conclude, the purpose of the IMAGINATION study (NCT05797610 2022-502102-32-00 WA43966) is to evaluate the efficacy, safety, and pharmacokinetics of RO7434656, a novel antisense oligonucleotide (ASO) inhibitor of complement factor B therapy, in participants with primary IgAN who are at high risk of progressive kidney disease despite optimized supportive care.

### 3.5. Fecal Microbiota Transplantation

Fecal microbiota transplantation (FMT) is the transfer of fecal samples from healthy donors into the gastrointestinal tract of patients with microbial dysbiosis. [62] Currently, FMT is recommended as the most effective therapy for recurrent Clostridium difficile infection [63].

New evidence also supports the role of FMT in other conditions, such as inflammatory bowel diseases, neurological disorders, metabolic syndrome, and autoimmune conditions [64].

The donor must be carefully selected. They must be a healthy individual with a balanced lifestyle, without chronic diseases or a family history of metabolic diseases, and laboratory tests should be performed to confirm the absence of ongoing disease [65].

Starting from the assumption that in patients with IgAN there is an alteration of the intestinal microbiota, new therapeutic strategies are emerging, such as the use of antibiotics or dietary implementation with prebiotics and/or probiotics, or FMT [66,67].

FMT has been proven to be effective in rebuilding the intestinal microecological balance by promoting the proliferation of beneficial bacteria while inhibiting the growth of damaging microorganisms. This equilibrium plays a pivotal role in preserving the integrity of the gut barrier and regulating the immune system [68].

Through addressing gut dysbiosis, FMT has the potential to mitigate the systemic inflammation that may play a role in the advancement of IgAN. In addition, FMT has the capacity to exert a beneficial impact on the recipient’s immune system [6].

An interventional study (clinical trial NCT03633864) is currently being conducted and intends to define the security and efficiency of FMT in IgAN subjects resistant to the standard therapy. This trial aims to evaluate the impact of FMT on the renal function, disease progression, and overall well-being of patients [69].

Lauriero et al. conducted a preclinical experiment using a humanized mouse model to investigate the effects of FMT on IgAN. They tested FMT from healthy subjects (HC), IgA progressor (P) patients, and non-progressor (NP) patients. The study found that metabolically active Firmicutes increased in the NP and P patients compared to the HC group, while Bacteroidetes were highest in HC. Mice given antibiotics alone showed a decreased urinary albumin to creatinine ratio (ACR), confirming that depleting the host microbiota protects against IgAN progression. Mice receiving FMT from healthy donors had a significantly reduced ACR at the end of FMT and two weeks later, suggesting that a healthy microbiota might delay IgAN progression. Hematuria did not significantly differ between groups [70].

As described by Zhao et al., FMT may be a therapeutic option in patients with IgAN unresponsive to conventional therapies. They treated a 48-year-old woman with FMT performed 40 times consecutively (200 mL daily, 5 days/week) and then a further 57 times (200 mL daily, 10–15 days/month) over the next five months. Another refractory IgAN was a 32-year-old woman that was treated with intensive fresh FMT, totaling 60 treatments in six months (200 mL daily, 10–15 days/month), and followed up for six months.

In both patients, the 24-hUP showed a decreasing trend, reaching less than half of baseline three months after the end of treatment. Serum albumin increased, and creatinine and eGFR remained stable during and after treatment. 

The alteration of the parameters of the gut microbiota before and after FMT was similar in the two patients: The phyla Proteobacteria and Verrucomicrobia were found in decreasing abundance, while the genus Prevotella was found in increasing abundance during FMT [6].

Zhi et al., reported the case of a patient with IgAN treated over the years with the maximum tolerated dosage of ACEi/ARB drugs, corticosteroid therapy, and antibiotics. Over time, the recurrences became more severe and more frequent, so they decided to treat him with FMT. The patient was monitored for half a year post-treatment and exhibited a notable decrease in the quantification of 24-hUP. Regarding the gut microbiota, the patient displayed enhanced diversity in the intestinal flora following FMT, which became closer in composition to a healthy subject. Some species of bacteria showed an increase in numbers, such as Bacteroides fragilis, Bacteroides ovatus, and Bacteroides stercoris, while others declined, such as Eggerthella lenta, Bilophila wadsworthia, and Escherichia coli. This was coupled with an elevation in Clostridium symbiosum, a bacterium whose prevalence is linked to increased renal ACE2 expression. ACE2 deficiency is associated with renal dysfunction, renal fibrosis, and various other kidney-related diseases [71].

Understanding the role of the gut microbiota in each chronic disease is essential for thinking about a personalized approach with FMT. Specifically, it is necessary to type the fecal microbiota of donors and recipients according to pathology and to understand how environmental factors (such as diet or medication intake) may influence the clinical response to FMT. All of this could obviously increase the therapeutic potential of FMT [65].

### 3.6. A Promising Experimental Treatment: Imoxin 

The role of interleukin 6 (IL-6) in the pathogenesis of IgAN has recently been the focus of attention.

In the case of acute pathological conditions such as infections or tissue damage, the expression of IL-6 is immediate and causes cell proliferation and differentiation; removal of the source of damage results in the cessation of IL-6 activation by ligand-induced internalization and degradation of gp130. [72] Dysregulation in this system provokes the development of several pathologies based on chronic inflammation and autoimmunity [73,74].

In a recent study, it was shown that the alteration of the VTRNA2–1/PKR/CREB/IL-6 pathway leads to an increase in IL-6 in patients with IgAN. The Vault RNA2-1 (VTRNA2-1) gene produces a polymerase III RNA transcript that acts as a direct inhibitor of protein kinase R (PKR), which plays a critical role in regulating cell growth. The VTRNA2-1 product prevents the autophosphorylation of protein kinase R (PKR), reducing its activity. In patients with IgAN, VTRNA2-1 is epigenetically downregulated by hypermethylation [75], and this causes increased activation of PKR by autophosphorylation (phosphoPKR; pPKR). pPKR causes phosphorylation of CREB Kinase (phosphoCREB; pCREB), which interacts with the IL-6 promoter and causes IL-6 levels to rise. 

To validate the increase in IL-6 dependent on PKR hyperactivation, the effect of the drug imoxin, also called imidazole–oxindole PKR inhibitor C16, which reduces the signaling of the PKR/CREB/IL-6 pathway, mimicking the natural activity of VTRNA2-1, was investigated. After 48 h following the administration of imoxin, both CREB phosphorylation and IL-6 secretion were significantly reduced in IgA patients. [76] In this context, the use of imoxin reduces the levels of angiotensin II, pCREB, reactive oxygen species (ROS), and advanced glycosylation products (AGEs) in treated mice [77,78].

Therefore, inhibition of PKR with imoxin not only reduces the burden of IL-6, a cytokine peculiarly implicated in the pathogenesis of IgAN, but also supports the reduction of factors that promote fibrosis and cell death. Therefore, it is plausible that it could slow the progression of refractory IgAN-induced renal disease.

## 4. Conclusions

Despite significant advances in the knowledge of the pathogenesis of IgAN, its therapy has not changed in the last 30 years [79]. Currently, approximately 30% of IgAN patients progress to ESKD [44,80].

In terms of therapeutic strategies, although IgAN is an immune-mediated disease [81], the use of immunosuppressive therapy remains controversial, recommended only for rapid-progressive forms of IgAN and secondary to supportive care. The latter is characterized primarily by the use of renin–angiotensin–aldosterone system blockade up to the highest tolerated dose—in order to control blood pressure and reduce proteinuria—and by lifestyle changes such as dietary salt restriction; smoking cessation; body weight control; and workouts [82]. Patients who present a persistent high risk of progressive ESKD, defined as IgAN patients with proteinuria > 0.75–1 g/day despite > 90 days of optimization supportive therapy, are candidates for glucocorticoid therapy. Despite the availability of these therapies, there are instances where certain patients exhibit a lack of response to treatment, and, although this definition is not provided in the KDIGO, some Authors define them as “refractory IgAN”. The scientific literature is limited on the topic, thereby limiting the scope of this review; nevertheless, this review highlights innovative therapeutic approaches to addressing “refractory IgAN”. 

## Figures and Tables

**Table 1:** Emerging therapeutic drugs and their corresponding ongoing clinical trials aimed at treating IgA nephropathy (IgAN).

Drug	Mechanism of Action	Route of Administration	Phase	ClinicalTrials.gov (Accessed on 1 December 2023) ID
Sparsentan	Selective antagonists of the angiotensin II receptor and endothelin A receptor	Oral	III	NCT03762850 (PROTECT)
Atrasentan	Antagonist of endothelin A receptor	Oral	III	NCT04573478 (ALIGN)
Hydroxychloroquine	TLR signaling inhibitor	Oral	II	NCT02942381
Blisibimod (AMG623)	Inhibits both soluble and membrane BAFF	Subcutaneous	II/III	NCT02062684
Sibeprenlimab (VIS649)	Humanized IgG2 monoclonal antibody that inhibits APRIL	Subcutaneous	III	NCT05248646
BION-1301	Monoclonal IgG4 antibody targeting APRIL	Intravenous infusion/subcutaneous	I/II	NCT03945318
Atacicept	Inhibits BAFF and APRIL	Subcutaneous	II/III	NCT04716231 (ORIGIN 3)
Telitacicept	Dual inhibitor of BAFF/APRIL	Subcutaneous	III	NCT05799287
Felzartamab (MOR202)	Humanized IgG1 monoclonal antibody against CD38	Intravenous infusion	II	NCT05065970 (IGNAZ)
Mezagitamab (TAK-079)	Anti-CD38 human IgG1 monoclonal antibody	Subcutaneous	I	NCT05174221
CCX168	Anti-C5a receptor antagonist	Oral	II	NCT02384317
Ravalizumab (ALXN1210)	Long-acting C5-blocking antibody	Intravenous infusion	II	NCT04564339
Cemdisiran (ALN-CC5)	Small interfering RNA-targeting C5	Subcutaneous	II	NCT03841448
Iptacopan (LNP023)	Selective C5a receptor inhibitor	Oral	II III	NCT03373461 NCT04578834 (APPLAUSE)
Pegcetacoplan (APL-2)	Prevents the cleavage and activation of C3	Subcutaneous	II	NCT03453619
Vemircopan (ALXN2050)	Factor D inhibitor	Oral	II	NCT05097989

## Data Availability

No new data were created or analyzed in this study. Data sharing is not applicable to this article.
